# Bio-priming with a consortium of *Streptomyces araujoniae* strains modulates defense response in chickpea against *Fusarium* wilt

**DOI:** 10.3389/fmicb.2022.998546

**Published:** 2022-09-08

**Authors:** Mohammad Tarique Zeyad, Pushpendra Tiwari, Waquar Akhter Ansari, Shiv Charan Kumar, Murugan Kumar, Hillol Chakdar, Alok Kumar Srivastava, Udai B. Singh, Anil Kumar Saxena

**Affiliations:** ICAR-National Bureau of Agriculturally Important Microorganisms, Mau, Uttar Pradesh, India

**Keywords:** bio-priming, chickpea, consortium, disease alleviation, *Streptomyces araujoniae*

## Abstract

Wilt caused by *Fusarium oxysporum* f. sp. *ciceris* (Foc) is one of the major diseases of chickpea affecting the potential yield significantly. Productivity and biotic stress resilience are both improved by the association and interaction of *Streptomyces* spp. with crop plants. In the present study, we evaluated two *Streptomyces araujoniae* strains (TN11 and TN19) for controlling the wilt of chickpea individually and as a consortium. The response of Foc challenged chickpea to inoculation with *S. araujoniae* TN11 and TN19 individually and as a consortium was recorded in terms of changes in physio-biochemical and expression of genes coding superoxide dismutase (SOD), peroxidase, and catalase. Priming with a consortium of TN11 and TN19 reduced the disease severity by 50–58% when challenged with Foc. Consortium primed-challenged plants recorded lower shoot dry weight to fresh weight ratio and root dry weight to fresh weight ratio as compared to challenged non-primed plants. The pathogen-challenged consortium primed plants recorded the highest accumulation of proline and electrolyte leakage. Similarly, total chlorophyll and carotenoids were recorded highest in the consortium treatment. Expression of genes coding SOD, peroxidase, and catalase was up-regulated which corroborated with higher activities of SOD, peroxidase, and catalase in consortium primed-challenged plants as compared to the challenged non-primed plants. Ethyl acetate extracts of TN11 and TN19 inhibited the growth of fungal pathogens *viz.*, *Fusarium oxysporum* f. sp. *ciceris*. *Macrophomina phaseolina, F. udum,* and *Sclerotinia sclerotiarum* by 54–73%. LC–MS analyses of the extracts showed the presence of a variety of antifungal compounds like erucamide and valinomycin in TN11 and valinomycin and dinactin in TN19. These findings suggest that the consortium of two strains of *S. araujoniae* (TN11 and TN19) can modulate defense response in chickpea against wilt and can be explored as a biocontrol strategy.

## Introduction

Chickpea is an important leguminous crop in India with a 70% share in global production and 71% share in the global area. Although India ranks first in the area under cultivation and total production, the country’s productivity (1,063 kg/ha) is much lower compared to many other countries (Lake and Sadras, 2014; Samriti et al., 2020). Lower productivity is attributed to poor adaptation of improved varieties and production technologies by farmers, drought during critical periods of crop growth (Muehe et al., 2019), and a series of biotic stressors like insect pests, and plant diseases (Ankati et al., 2021). Globally, crop growth and production have always been at risk from plant diseases, which obstruct physiological processes like photosynthesis, cell division, water transport, growth, and development. According to estimates, these phytopathogens are responsible for roughly 12.5% of all crop losses worldwide. The fungi are the most harmful phytopathogens, and they can cause 65% loss in plants (Fisher et al., 2012). As per the food and agriculture organization corporate statistical database (FAOSTAT), at the global level, most of the economically important crops are affected by fungi. Numerous fungal diseases have increased in frequency under the current climate change scenario. (Garcia-Solache and Casadevall, 2010). To ensure a steady supply of food for the growing global population, management of these fungal diseases is essential.

Among the different biotic stressors experienced by chickpea during its growth phase, wilt caused by *Fusarium oxysporum* f. sp. *ciceris* (Foc) can lead to high yield loss to complete crop failure under favorable conditions. Foc is both soil-borne and seed-borne and can survive as long as 6 years in the soil even without a susceptible host (Nikam et al., 2007). The use of resistant varieties, one of the most economical and practical solutions for managing fungal diseases, did not result in the successful management of Foc due to the availability of eight pathogenic races (Nikam et al., 2007; Ankati et al., 2021). Other management practices like soil solarization and early sowing have all gone in vain (Ankati et al., 2021). Although some successes have been achieved in controlling this disease using chemical fungicides (Jamil and Ashraf, 2020), the use of chemical fungicides can lead to environmental issues. Under such a scenario it is only pragmatic to look for safe and eco-friendly measures of disease management, i.e., biological control. A variety of commercial formulations based on *Trichoderma* spp. and *Pseudomonas fluorescens* are available in the market to control Foc. There are many reports available on the biocontrol of Foc using different bacterial and fungal agents like *Bacillus* spp. *Pseudomonas* spp. *Trichoderma* spp. *Paenibacillus polymyxa* and non-pathogenic Foc (Jiménez-Díaz et al., 2015). Over the last decade, there is an increased interest in the search for new biocontrol agents with better efficiency, adaptation, and colonization. Due to their diversity and potential to create unique antibiotics, antifungal metabolites, and extracellular enzymes, actinomycetes are the most economically and biotechnologically valuable prokaryotes (Mohite et al., 2019; Savi et al., 2019). Actinomycetes inhabiting various rhizosphere soils have also been reported to produce active biomolecules which promote plant growth like phytohormones, siderophores, iron chelators, and organic acids (Anwar et al., 2016). Various actinomycetes, mainly belonging to the *Streptomyces* genus have been reported as antifungal agents that can prevent the growth of plant pathogenic fungi (Palla et al., 2018). Apart from these, many *Streptomyces* showed antibacterial (Bo et al., 2018), nematicidal (Park et al., 2020), antioxidant, antiviral, and anticancer activities (Fahmy et al., 2022). All these functions are powered by the production and release of hydrolytic enzymes, competition, antibiotic production, and the formation of cyanogenic chemicals. A variety of *Streptomyces* species produce volatile organic compounds (VOCs) with potent antifungal properties (Qi et al., 2019). Due to their potential to synthesize different kinds of antimicrobial compounds, *Streptomyces* spp. can be potential candidates for new biocontrol agents.

Microbial bio-priming, which is an adaptive technique to increase the defense capabilities of plants, results in enhanced resistance/tolerance to stress, and a more exacerbated defense response to stress (Aamir et al., 2019). Defense response of plants against pathogen invasion includes activation of reactive oxygen species (ROS) system, enhanced deposition of suberin and lignin for strengthening of cells at the site of infection, and expression of pathogenesis-related (PR) proteins (Pusztahelyi et al., 2015), which highlights the function of the systemic acquired resistance (SAR) pathway (Gharbi et al., 2017). β-1, 3-glucanases, and endochitinases are two important PR proteins, differentially expressed in many plant species infected by fungal pathogens (Ebrahim et al., 2011; Balasubramanian et al., 2012). The phenylpropanoid pathway is activated in actinobacteria-mediated bio-priming, characterized by the release of numerous antimicrobials. This process not only inhibits the infection and spread of pathogens but also provides tolerance to various abiotic stressors. Numerous studies have shown that treating plants with beneficial microorganisms improves their ability to fend off infections and other abiotic stresses by activating the ROS system (Brotman et al., 2013; Fu et al., 2017). The role of *Streptomyces* spp. mediated bio-priming in Foc-challenged plants concerning signaling pathways and molecular mechanisms has not been explored. Hence the present investigation has been planned to better understand defense signaling, various physio-biochemical alterations, and changes in gene expression due to bio-priming with *Streptomyces araujoniae*.

In our earlier studies on different actinobacterial strains, we reported several species of *Streptomyces* antagonistic against Foc, *F. udum*, *Macrophomina phaseolina,* and *Sclerotium rolfsii*. We selected two strains of *Streptomyces araujoniae* TN11 and TN19 based on their *in vitro* antagonistic potential against the four pathogens. They were explored for their potential as biocontrol agents against Foc as individuals and as a consortium *via in planta* assays. Additionally, we have analyzed the antifungal compound profile of these two *Streptomyces* strains using liquid chromatography and mass spectrometry (LC–MS).

## Materials and methods

### Actinobacterial strains and fungal plant pathogens

Two strains of actinobacteria *Streptomyces araujoniae* TN11 (NAIMCC-B-02868) and *S*. *araujoniae* TN19 (NAIMCC-B-02870) and four phytopathogenic fungi *viz.*, *Macrophomina phaseolina* (NAIMCC-F-01261), *Fusarium oxysporum* f. sp. *ciceris* (NAIMCC-F-02001), *Fusarium udum* (NAIMCC-F-01103), and *Sclerotinia sclerotiorum* (NAIMCC-F-03341) were procured from the National Agriculturally Important Microbial Culture Collection (NAIMCC; WDCM Reg. No. 1060), Mau, India. Actinobacterial strains were revived by growing on starch casein agar (SCA) media (HiMedia, India) for 3–5 days at 30°C. They were chosen from among the 65 strains of actinobacteria (isolated from Tamil Nadu) based on their antagonistic activity against the Foc (data submitted elsewhere). Fungal strains were grown on potato dextrose agar (PDA) media for 3–5 days at 28 ± 2°C.

### Pathogen inoculum preparation and pathogenicity test

*Fusarium oxysporum* f. sp. *ciceris* (Foc) was grown on PDA (Hi-media, India) for 3–5 days at 28 ± 2°C. The fully grown culture was suspended in sterile distilled water; filtered using a muslin cloth to collect the spores. The spore suspension was diluted to contain 10^6^ spores per mL (Taylor et al., 2013). A pathogenicity test was conducted to confirm the virulence and to demonstrate the pathogen’s participation in the development of vascular wilt disease symptoms. The conventional root dip technique was used to inoculate 20 days old healthy plants (Nirmaladevi et al., 2016). The healthy seedlings of chickpea (Var. Pusa 362) maintained in pots with sterilized soil were gently uprooted from their pots without damaging their root systems, shaken to remove any attached dirt particles, then gently rinsed under sterile water. The apex root part (approximately 1 cm) was trimmed using a sterile scissor and it was dipped in the spore suspension of Foc for 30 min. The treated plants were then planted in small pots (d: 9 cm, surface sterilized with mercuric chloride) containing a 2:1 mixture of sterilized soil and sand. Pots were kept in a greenhouse for L: D:: 16:8 h, with 22–24°C (day) and 16–18°C (night) and humidity of 60%. The symptoms of vascular wilt infection appeared 15–20 days after pathogen inoculation. A parallel control is maintained by dipping the healthy seedlings in sterile distilled water to confirm the vascular wilt infection is only due to the spore suspension of Foc.

### Actinobacterial inoculum preparation

Compatibility between TN11 and TN19 was checked on starch casein agar plates and was found compatible. The strains were grown in starch casein broth in an incubator shaker (Jeiotech, Korea) for 5 days at 30°C with continual shaking (120 rpm). After 5 days, the fully grown cultures were subjected to centrifugation at 6000 rpm for 10 min. Cell pellets were then washed with sterile distilled water and resuspended in phosphate buffer (0.1 M, pH-7.0) to a final concentration of Abs >1.0 at 600 nm. A consortium of TN11 and TN19 was prepared by mixing these two actinobacterial suspensions in equal volumes (Chukwuneme et al., 2020).

### Pot trial

A pot experiment was performed to evaluate the potential of selected strains as individuals and as a consortium against Foc wilt of chickpea in greenhouse conditions with temperatures 22–24°C (day) and 16–18°C (night) and humidity of 60%. The potting mixture of sand and soil in the ratio of 2:1 was sterilized in an autoclave at 121°C for 1 h, thrice (on three consecutive days). The sterilized potting mixture was then filled in pots (d: 9 cm) at 4 kg/pot. Foc pathogen grown on sterilized sorghum seeds was mixed with the potting mixture at 5 g/kg soil. Fresh and healthy seeds of chickpea (variety Pusa 362) were surface sterilized with 0.1% mercuric chloride for 3 min, followed by treatment with 70% ethanol for 1 min. They were then subjected to washing with sterile distilled water twice and dried under aseptic conditions (Jain et al., 2012). For bio-priming with actinobacterial cultures and their consortium, 15 gm of surface sterilized seeds were soaked in 30 ml of actinobacterial suspension and their consortium for 30 min based on the treatment. The control seeds were soaked in sterilized phosphate buffer for 30 min. Treated seeds were then planted in pots (4 seeds per pot), 48 h after inoculation with the pathogen. A total of five treatments were taken up with five replications each. Treatments include control (no actinobacteria and no Foc), Foc alone (Foc), Foc + TN11, Foc + TN19, Foc + TN11 + TN19 (Foc + consortium of TN11 and TN19). The experiment was carried out up to 50 days after sowing; disease appearance started five weeks after sowing.

### Disease severity index

A rater’s assessment of the disease severity was used to calculate the disease severity index (DSI), expressed as a percentage (Chiang et al., 2017). The disease severity index is calculated as follows:


DSI(%)=[sum(c×s)]/[(n)×(md)]×100.

*c*, class frequency; *s*, score of rating class; *n*, total number of plants; md, maximal disease index.

### Morphological parameters

All the parameters were observed 50 days after sowing. Plants were separated from the soil with their roots intact and the shoot length and root length were measured. Parts of the root and shoot were separated, to record the fresh mass of the shoot and root. The dry mass of shoot and root was recorded after drying the plant portions for 48 h at 80°C in an oven. The ratio of dry mass to the fresh mass of root and shoot was estimated (Ansari et al., 2018). Stem width and number of leaves were also recorded.

### Relative water content and electrolyte leakage

Relative water content (RWC) and electrolyte leakage (EL) were measured (Khare et al., 2010) and expressed in percentage. Twelve leaf discs were weighed to obtain the fresh mass (F). Leaf discs were then soaked in water for 6 h, surface-dried, and weighed to obtain the turgid mass (T). The soaked leaf discs were then oven-dried for 24 h at 80°C and weighed to get dry mass (D). RWC was calculated using the following formula,


RWC=[(F−D)/(T−D)]×100.

Ten leaf discs were placed in 30 ml of double-distilled water and kept at 28°C for 4 h. Conductivity was measured after four hours (a). The contents were then autoclaved for 30 min at 121°C and conductivity was measured again (b). The EL was determined using the formula,


EL=(a/b)×100.

### Hydrogen peroxide, lipid peroxidation, and proline content

For estimation of H_2_O_2_, 0.2 gm of leaf samples were homogenized in 5 ml of sodium phosphate buffer (50 mM; pH 6.5) and centrifuged for 20 min at 6,000× *g*. Three milliliters of the resultant supernatant was then mixed with 1 ml of titanium sulfate (0.1% w/v) dissolved in 20% H_2_SO_4_. The mixture was then subjected to centrifugation for 15 min at 6,000× *g*. The supernatant was collected and the absorbance was measured at 410 nm (UV-vis 1601 Shimadzu, Japan). The amount of H_2_O_2_ was then expressed as μm g^−1^ fresh weight (Jana and Choudhuri, 1981).

For estimation of lipid peroxidation, 0.4 g of leaf tissue was homogenized in four mL trichloroacetic acid reagent comprising 0.1% trichloroacetic acid, 0.5% butylated hydroxytoluene, and 1% polyvinylpyrrolidone. The mixture was subjected to centrifugation for 20 min at 6,000× *g*. The resultant supernatant (2.5 ml) was mixed with 0.5% thiobarbituric acid and 20% trichloroacetic acid and boiled for 30 min. After 30 min the mixture was centrifuged for 20 min at 6,000× *g*. The supernatant’s absorbance at 532 nm was measured, and nonspecific turbidity correction was performed by subtracting the absorbance at 600 nm. Lipid peroxidation was expressed as μM malondialdehyde g^−1^ fresh weight (Heath and Packer, 1968).

To measure the proline concentration, leaf samples (0.2 gm) were crushed in 3% sulfosalicylic acid and centrifuged for 10 min at 13,000× *g*. The supernatant (0.5 ml) was incubated at 100°C for 60 min with 0.5 ml each of ninhydrin reagent (freshly prepared) and glacial acetic acid. After 60 min one mL of toluene was added to the mixture and the absorbance was recorded at 520 nm (Bates et al., 1973). The amount of proline was expressed as μg g^−1^ fresh weight.

### Photosynthetic pigments

To measure the chlorophyll and carotenoid content, 0.3 g of fresh leaf samples were homogenized in 80% chilled acetone. The mixture was centrifuged for 10 min. at 8000× *g* and the absorbance (UV-vis 1601 Shimadzu, Japan) of the supernatant was recorded at 663 nm (A_663_), 645 nm (A_645_), and 470 nm (A_470_). The following formulae were used to calculate the chlorophyll and carotenoid contents expressed as mg g^−1^ fresh weight,


$$\begin{array}{l} \mathrm{Chlorophyll}\;a=\left[\left(12.7\times A_{663}\right)-\left(2.69\times A_{645}\right)\right];\\ \mathrm{Chlorophyll}\;b=\left[\left(22.9\times A_{645}\right)-\left(4.68\times A_{663}\right)\right];\\ \mathrm{Carotenoids}=\left[\left(1,000\times A_{470}\right)-\left(\begin{array}{l} 3.27\times \mathrm{Chlorophyll}\;a\\ +\mathrm{Chlorophyll}\;b \end{array}\right)\right]/227. \end{array}$$

### Determination of antioxidant enzyme activity

For superoxide dismutase (SOD) assay fresh leaf sample (0.2 gm) were crushed in 5 ml potassium phosphate buffer (100 mm; pH 7.5) containing EDTA (0.5 mm), Triton X-100 (0.1%) and PVP (2%). The mixture was subjected to centrifugation for 15 min. at 15000 under 4°C. SOD activity was measured from the supernatant in an assay mixture (3 ml) containing sodium carbonate–bicarbonate buffer (50 mm; pH 9.8), EDTA (0.5 mm), and epinephrine (0.6 mm). Absorbance was recorded at 470 nm (UV-vis 1601 Shimadzu, Japan) to record adrenochrome generation (Shah et al., 2001). SOD was expressed as U (mg protein)^−1^.

For catalase activity, fresh leaf sample (0.2 gm) was crushed in 5 ml Tris-NaOH buffer (50 mm; pH 8.0), containing EDTA (0.5 mm), PVP (2%), and Triton X-100 (0.5%). The mixture was subjected to centrifugation for 15 min at 15000 under 4°C. Catalase activity was measured from the supernatant in an assay mixture (1.5 ml) containing 1 ml potassium phosphate buffer (100 mm; pH 7.0), 0.4 ml H_2_O_2_ (200 mm), and 0.1 ml enzyme extract. Absorbance (UV-vis 1601 Shimadzu, Japan) was recorded at 240 nm (Rai et al., 2012). Catalase activity was expressed as μmol of H_2_O_2_ oxidized (mg protein)^−1^ min^−1^.

For Peroxidase (POD) activity fresh leaf samples (0.2 gm) were crushed in five mL of sodium phosphate buffer (60 mm; pH 7.0). The mixture was subjected to centrifugation for 15 min at 15000 rpm under 4°C. POD activity was measured from the supernatant in an assay mixture (2 ml) containing guaiacol (200 μl) H_2_O_2_ (50 μl), enzyme extract (50 μl) phosphate buffer (1.7 ml). Absorbance (UV-vis 1601 Shimadzu, Japan) was measured at 470 nm (Shah et al., 2001). The activity was expressed as H_2_O_2_ reduced (mg protein)^−1^ min^−1^.

### RNA extraction and cDNA synthesis

Relative expression of genes coding antioxidant enzymes was determined quantitatively in leaf tissues from all treatments. Total RNA was isolated using TRIZOL reagent (Invitrogen) following the instruction manual. The quality of RNA was confirmed through gel electrophoresis (1.0% agarose gel prepared in DEPC treated water). Nanophotometer (Implen, CA, United States) was also used to evaluate the quantity, purity, and integrity of RNA. cDNA synthesis was carried out from the RNA (1 μg) employing the Iscript™ cDNA synthesis kit (Bio-Rad Laboratories United States) as following the instruction manual.

### Real-time quantitative PCR analysis

Quantitative real-time PCR (qRT-PCR) assay was carried out in an iQ5 thermocycler (BioRad Laboratories, United States). PCR reactions were carried out with a reaction mixture containing 2 μl of cDNA template (20 ng), 1 μl each of primers (0.2 μM) of *SOD*, *POD,* and *CAT* (Supplementary Table 1), and 10 μl of iQ-SYBR Green Supermix (Bio-Rad, United States) and an appropriate volume of Milli-Q water to a final volume of 20 μl. The qRT PCR program used was; initial denaturation for 8 min at 95°C followed by 35 cycles of denaturation at 95°C for 20 s, annealing at 60°C for 30 s, and extension at 72°C for 30 s. ACTIN (chickpea) gene was used as a reference because of its constant and steady expression (Karkute et al., 2019). The Δ^Ct^ value was calculated by subtracting the Ct values of the housekeeping gene (ACTIN) from the target gene. The relative quantification was examined using Livak and Schmittgen’s (2001) 2^−ΔΔCT^ method. The Ct value concerning the transcript level of the ACTIN gene was then normalized as an internal control.

### Cell-free extracts of actinobacteria and their antifungal assay

Actinobacterial strains were grown as broth culture in starch casein broth for preparation of mother culture. One liter of broth culture is raised with 5% mother inoculum and grown with continuous agitation at 120 rpm for 10 days at 30°C. After incubation was complete, the obtained culture broth was centrifuged at 8000 rpm for 20 min at 4°C. The supernatant was collected and metabolites were extracted with an equal volume of different organic solvents *viz.*, ethyl acetate, n-hexane, and dimethyl sulfoxide (SRL, India). After extraction, excess solvents were evaporated in a rotary vacuum evaporator (Hahnshin, Korea) and the resultant metabolites were dissolved in the respective solvents to a final volume of 2 ml. Cell-free extracts (supernatant) were collected and subjected to antagonistic activity against the pathogenic fungi by amending the extracts at 1% in the growth media just before plating. Metabolites extracted using different solvents were tested for their ability to control selected phytopathogenic fungi by employing the well diffusion assay. The assay is carried out on a media containing PDA and SCA in the ratio of 1:1. From a fully grown fresh culture, 6 mm fungal discs were placed in the center of PDA/SCA media plates. Wells (d: 4 mm) were made in the plates at four places on the plate around the fungus disc. Solvent extracts (20 μl) were placed in each well with solvent as the negative control. The plates were incubated at 28 ± 2°C for 3–5 days. Growth inhibition of fungal pathogen was recorded regularly. The percent growth inhibition of pathogen was recorded for both cell-free extracts and organic solvent extracts (Shahid et al., 2021).

### LC–MS analysis of ethyl acetate extract

Liquid chromatography-mass spectrometry was carried out at Central Instrumentation Facility, South Campus, Delhi University (DU), India. The metabolites were identified using Thermo Fisher Scientific Compound Discoverer 2.2. The data were analyzed, and it was used to predict chemical formulae and identify the peaks. mZCloud; an Advanced Mass Spectral Database^2^ and Chemspider database^3^ were used (Shahid et al., 2021).

### Statistical analysis

The experimental data were analyzed by One-Way analysis of variance (ANOVA) using SPSS software (V-16). Duncan’s Multiple Range Test (DMRT) at *p* < 0.05 was used to further examine treatments and rank them. Analyzed data were presented as mean ± SE.

## Results

### Chickpeas disease control

The disease severity index (DSI) was calculated as a measure of disease control in different treatments. DSI of Foc, Foc + TN11, Foc + TN19, and Foc + TN11 + TN19, treated plants were 100, 50, 44.44 and 41.66%, respectively. Hence, disease severity is reduced by 50, 55.56, and 58.33% by priming with TN11, TN19, and the consortium of TN11 + TN19 respectively.

### Growth promotion

Priming chickpea seeds with *S. araujoniae* strains individually and as a consortium resulted in the profuse growth of the chickpea plants in the presence of pathogens, exhibiting a significant increase in the morphological attributes *viz.*, shoot and root length, plant height, the thickness of stem, and the number of leaves. Effects of different treatments on the shoot and root length, plant height, stem width, and the number of leaves are presented in Table 1. In the presence of the Foc challenge, priming chickpea seeds with the consortium of TN11 + TN19 recorded the highest values for shoot length, root length, plant height, stem width, and the number of leaves while Foc challenged plants without any priming exhibited the lowest values. Priming with individual strains of *S. araujoniae* also showed a significant increase in shoot and root length, plant height, stem width, and the number of leaves over the unprimed Foc challenged plants. But the values recorded were lower than that of the consortium treatment.

**Table 1:** Shoot length, root length, plant height, stem width, and the number of leaves of chickpea plants under unprimed control, Foc challenged, Foc challenged + TN11 primed, Foc challenged + TN19 primed, and Foc challenged + TN11 + TN19 primed, conditions.

Treatment	Shoot length (cm)	Root length (cm)	Plant height (cm)	Stem width (cm)	No. of leaves
Control	30.0 ± 2.28^b^	5.03 ± 0.28^a^	35.0 ± 4.29^ab^	1.92 ± 0.09^b^	793 ± 64.2^ab^
Foc only	22.4 ± 1.73^d^	3.82 ± 0.21^c^	26.2 ± 1.35^c^	1.54 ± 0.04^d^	572 ± 28.9^d^
Foc + TN11	28.2 ± 1.54^bc^	4.94 ± 0.33^a^	33.1 ± 1.85^b^	1.81 ± 0.17^c^	712 ± 23.4^c^
Foc + TN19	28.5 ± 0.95^bc^	4.71 ± 0.49^ab^	33.2 ± 1.62^b^	1.83 ± 0.13^c^	588 ± 47.3^d^
Foc + TN11 + TN19	33.5 ± 1.69^a^	5.13 ± 0.24^a^	38.3 ± 1.55^a^	2.13 ± 0.11^a^	812 ± 39.1^a^

The data are the mean of three replicates ± standard error, within each column, values followed by the same letter are not significantly different (*p* ≤ 0.05) according to Duncan’s multiple range test.

Effects of different treatments on the shoot and root fresh weight, shoot and root dry weight, and the ratio of dry to fresh weight of shoot and root are presented in Table 2. They are found to show significant differences among the treatments. In Foc challenged treatments priming with consortium has shown the highest values of shoot fresh weight, root fresh weight, and root dry weight while priming with TN11 recorded the highest shoot dry weight. The ratio of dry to fresh weight of shoot was highest in unprimed Foc challenged plants (0.416) followed by Foc + TN11 (0.394), although both treatments are statistically at par, while it was least in Foc + TN11 + TN19 and at par with Foc + TN19 and control (non-challenged and unprimed) treatments. Similarly, the ratio of dry to fresh weight of root was again highest in unprimed Foc challenged plants (0.366), while it was least in Foc + TN11 + TN19 (0.165).

**Table 2:** Shoot fresh weight (SFW), shoot dry weight (SDW), root fresh weight (RFW), root dry weight (RDW), SDW/SFW ratio, RDW/RFW ratio, relative water content (RWC), and electrolyte leakage (EL), of chickpea plants under unprimed control, Foc challenged, Foc challenged + TN11 primed, Foc challenged + TN19 primed, and Foc challenged + TN11 + TN19 primed, conditions.

Treatment	SFW (gm)	SDW (gm)	RFW (gm)	RDW (gm)	SDW/SFW	RDW/RFW	RWC (%)	EL (%)
Control	12.8 ± 0.883^a^	3.42 ± 0.283^a^	1.5 ± 0.08^c^	0.319 ± 0.022^b^	0.267 ± 0.012^b^	0.213 ± 0.014^b^	70.59 ± 6.32^ab^	30.77 ± 2.68^cd^
Foc only	2.2 ± 0.112^e^	0.915 ± 0.091^d^	0.7 ± 0.05^f^	0.256 ± 0.011^c^	0.416 ± 0.024^a^	0.366 ± 0.029^a^	50.10 ± 3.82^e^	61.79 ± 6.72^a^
Foc + TN11	6.5 ± 0.273^d^	2.56 ± 0.133^b^	1.8 ± 0.11^b^	0.319 ± 0.025^b^	0.394 ± 0.029^a^	0.177 ± 0.014^c^	60.63 ± 2.46^d^	38.46 ± 1.68^b^
Foc + TN19	7.8 ± 0.521^bc^	2.32 ± 0.114^bc^	0.8 ± 0.03^e^	0.165 ± 0.011^d^	0.297 ± 0.025^b^	0.206 ± 0.007^b^	67.90 ± 5.78^bc^	38.89 ± 2.23 ^b^
Foc + TN11 + TN19	8.3 ± 0.392^b^	2.17 ± 0.153^c^	3.8 ± 0.24^a^	0.627 ± 0.041^a^	0.261 ± 0.017^b^	0.165 ± 0.009^d^	74.11 ± 8.24^a^	33.33 ± 1.88^c^

The data are mean of three replicates ± standard error, within each column, values followed by the same letter are not significantly different (*p* ≤ 0.05) according to Duncan’s multiple range test.

### Relative water content and electrolyte leakage

Significant variation among the treatments was observed for RWC and EL (Table 2). RWC was highest in Foc challenged plants receiving priming with the consortium of TN11 + TN19 (74.11%) followed by control plants (unprimed and non-challenged), while the lowest RWC was recorded in Foc challenged unprimed plants (50.10%). EL was highest in unprimed Foc challenged plants (61.79%) followed by Foc challenged plants primed with TN19 (38.89%), Foc challenged plants primed with TN11 (38.46%) and Foc challenged plants primed with the consortium of TN11 + TN19 (33.33%). Among Foc challenged plants treatments primed with TN11 + TN19 recorded the lowest EL which is statistically on par with control plants.

### Quantitative estimation of hydrogen peroxide, lipid peroxidation, and proline

**Figure 1:**
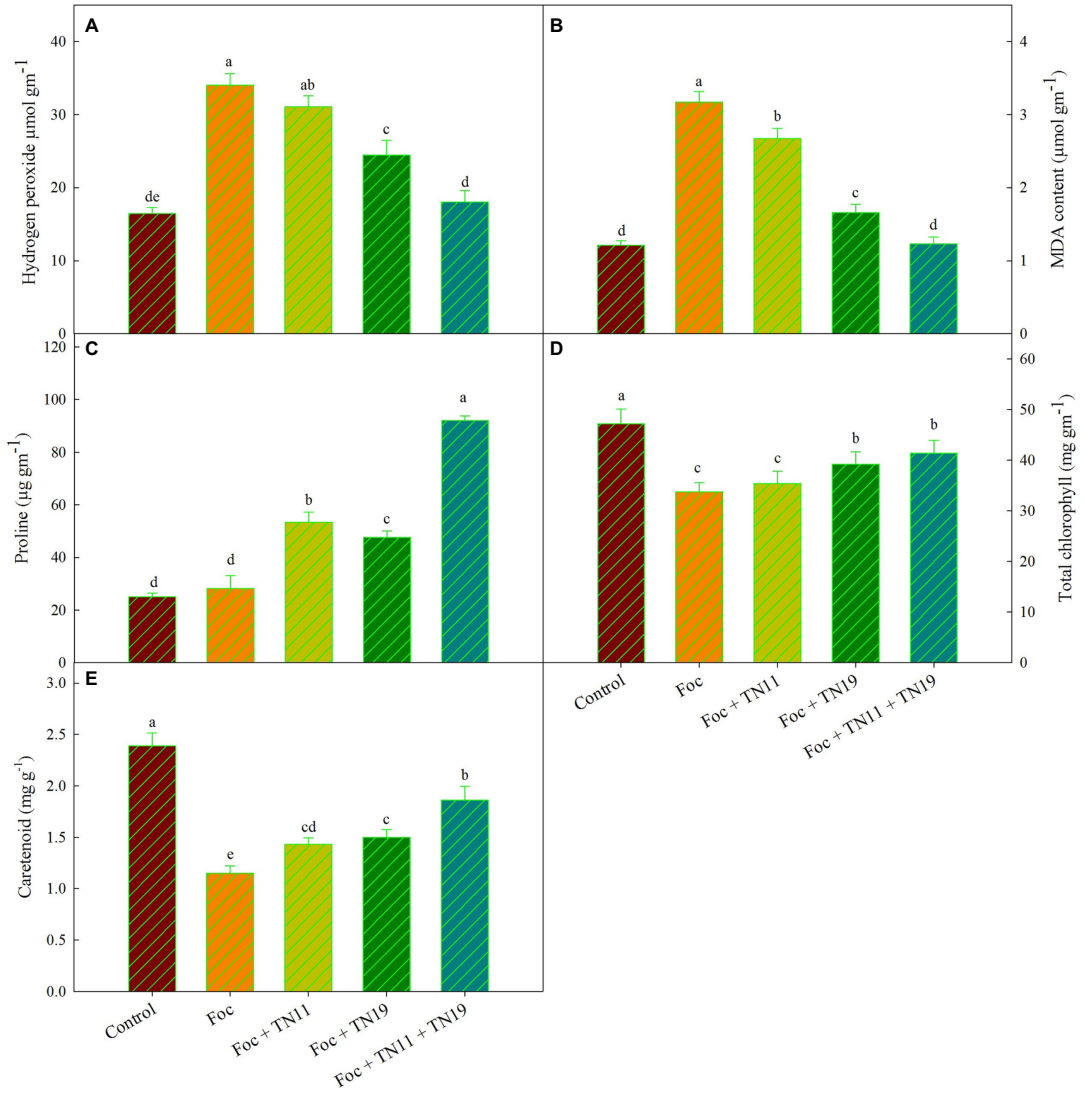
**(A)** Hydrogen peroxide, **(B)** lipid peroxidation (MDA content), **(C)** proline, **(D)** total chlorophyll, **(E)** carotenoids, in chickpea leaves under unprimed control, Foc challenged, Foc challenged + TN11 primed, Foc challenged + TN19 primed, and Foc challenged + TN11 + TN19 primed conditions. The data are the mean of three replicates ± standard error, within each graph, values followed by the same letter are not significantly different (*p* ≤ 0.05) according to Duncan’s multiple range test.

The amount of H_2_O_2_ generation was recorded significantly higher in Foc challenged plants and reduced in Foc challenged plants receiving priming with TN11, TN19, and the consortium of TN11 and TN19. Among the Foc challenged plants treatments primed with the consortium of TN11 + TN19 recorded the lowest accumulation of H_2_O_2_ (Figure 1A). There was 47.02% reduced accumulation of H_2_O_2_ in Foc challenged plants receiving consortium of TN11 + TN19 compared to unprimed plants.

Lipid peroxidation was quantified in terms of MDA generation. MDA was significantly higher in Foc challenged plants (3.17 μmol g^−1^ (FM)) followed by Foc + TN11 (2.67 μmol g^−1^ (FM)), Foc + TN19 (1.66 μmol g^−1^ (FM)) and Foc + TN11 + TN19 (1.23 μmol g^−1^ (FM)). MDA generation in Foc + TN11 + TN19 was statistically on par with control treatment that did not receive any pathogen and priming. Compared to Foc challenged plants, the percent reduction in MDA generation was 15.78, 47.64, and 61.20%, respectively in Foc challenged plants primed with TN11, TN19, and the consortium of TN11 and TN19 (Figure 1B).

Similarly, accumulation of proline was highest (92.07 μg^−1^ FM) in Foc + TN11 + TN19 followed by Foc + TN11 (53.42 μg^−1^ FM), Foc + TN19 (47.67 μg^−1^ FM) and Foc challenged plants (28.20). Proline accumulation in Foc + TN11 + TN19 was statistically on par with control plants (Figure 1C). Compared to Foc challenged plants percent increase in proline accumulation was 89.43, 69.04, and 226.5%, respectively in Foc challenged plants primed with TN11, TN19, and the consortium of TN11 and TN19 (Figure 1C).

### Photosynthetic pigments

Total chlorophyll content in leaves of all the treatments along with control was recorded, and it was found maximum (47.23 mg g^−1^) in control unprimed plants, followed by Foc + TN11 + TN19 (41.37 mg g^−1^), Foc + TN19 (39.23 mg g^−1^), Foc + TN11 (35.42 mg g^−1^) and Foc challenged plants (33.73 mg g^−1^). A non-significant difference in total chlorophyll content was recorded between Foc challenged plants and Foc challenged plants primed with TN11 (Figure 1D). Carotenoid content was maximum (2.39 mg g^−1^) in control unprimed plants, while it was lowest (1.15 mg g^−1^) in Foc challenged plants (Figure 1E). Compared to Foc challenged plants percent increase in carotenoid content was of 61.74% in Foc + TN11 + TN19.

### Antioxidative enzymes

#### SOD, POD, and CAT activity

**Figure 2:**
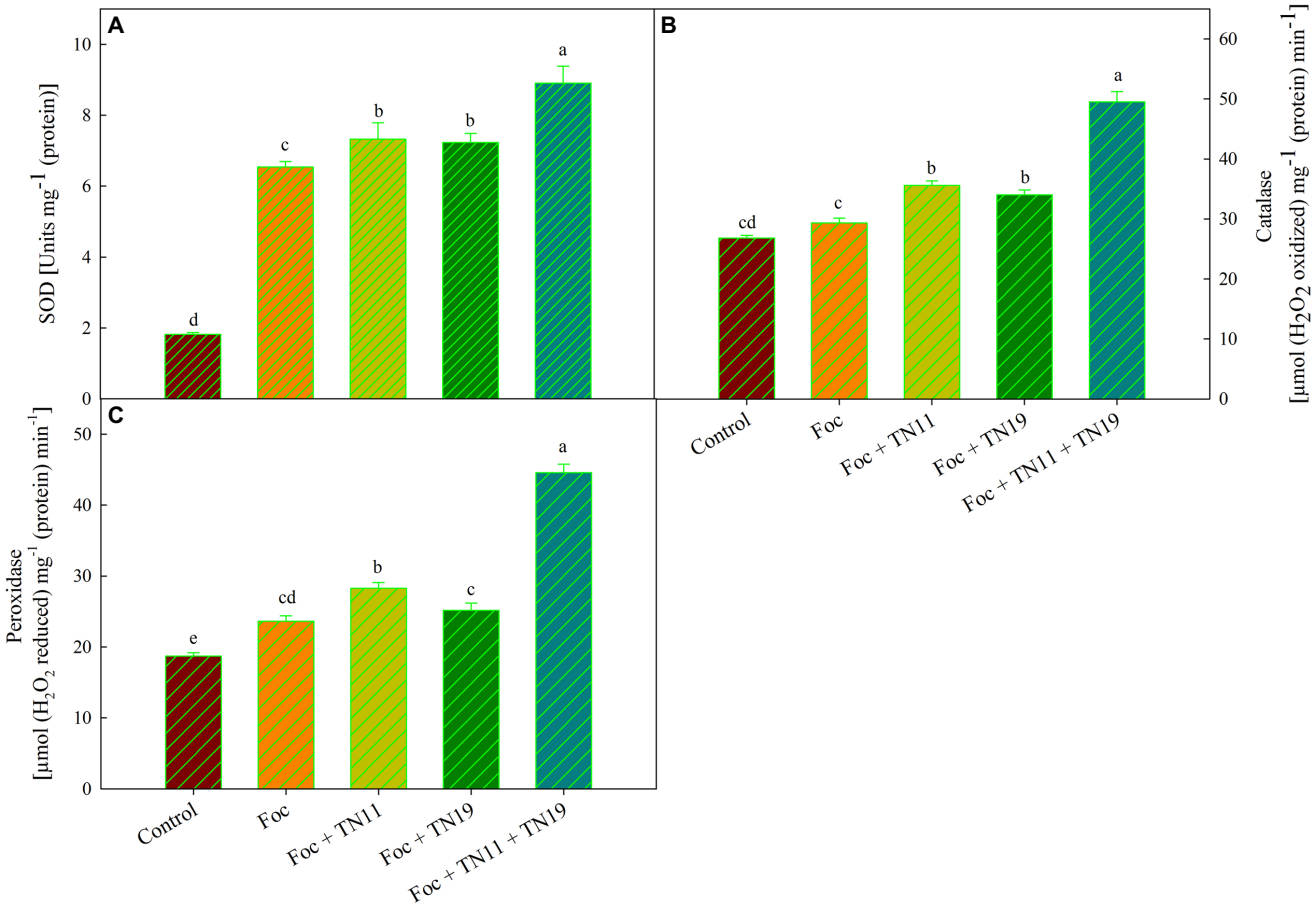
**(A)** Superoxide dismutase (SOD), **(B)** catalase (CAT), **(C)** peroxidase (POD), in chickpea leaves under unprimed control, Foc challenged, Foc challenged + TN11 primed, Foc challenged + TN19 primed, and Foc challenged + TN11 + TN19 primed. The data are the mean of three replicates ± standard error, within each graph, values followed by the same letter are not significantly different (p ≤ 0.05) according to Duncan’s multiple range test.

SOD activity was maximum (8.9 U mg^−1^ (protein)) in Foc + TN11 + TN19 treated plants, while it was 7.3 U mg^−1^ (protein) and 7.2 U mg^−1^ (protein) and 6.54 U mg^−1^ (protein) in Foc + TN11 and Foc + TN19 and Foc challenged plants, respectively (Figure 2A). CAT activity was minimum; 29.33 μmol (H_2_O_2_ reduced) min^−1^ mg^−1^ (protein) in Foc challenged plants, and a significant increase in catalase activity was recorded in all the treatments receiving priming. CAT activity recorded in Foc + TN11, Foc + TN19, and Foc + TN11 + TN19, respectively, were 1.21-, 1.16- and 1.69-folds of the same in Foc challenged unprimed plants (Figure 2B). Similarly, POD activity of Foc + TN11, Foc + TN19 and Foc + TN11 + TN19, respectively were 1.20-, 1.06- and 1.89-folds, the same recorded in Foc challenged unprimed plants (Figure 2C).

### Gene expression analysis

**Figure 3:**
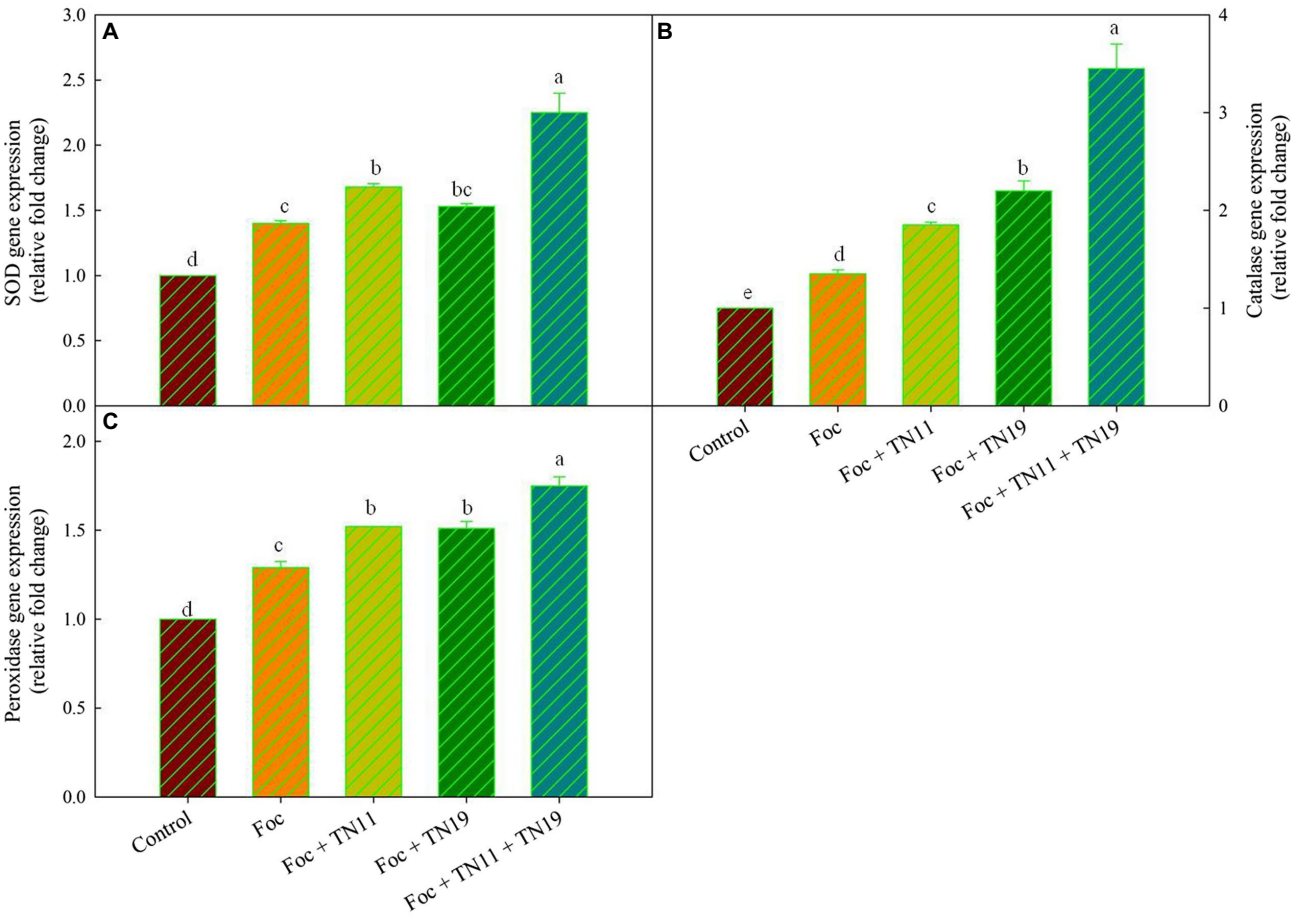
Relative expression of **(A)** superoxide dismutase (SOD), **(B)** catalase (CAT), **(C)** peroxidase (POD), gene in chickpea leaves under unprimed control, Foc challenged, Foc challenged + TN11 primed, Foc challenged + TN19 primed, and Foc challenged + TN11 + TN19 primed, conditions. The data are the mean of three replicates ± standard error, within each graph, values followed by the same letter are not significantly different (*p* ≤ 0.05) according to Duncan’s multiple range test.

It was found that an upregulated expression of *SOD*, *POD*, and *CAT* gene was recorded in Foc + TN11, Foc + TN19, and Foc + TN11 + TN19 treatments (Figure 3). Compared to control 1.40-, 1.68-, 1.53-, and 2.25- folds enhanced SOD expression were recorded in Foc, Foc + TN11, Foc + TN19 and Foc + TN11 + TN19 treatments, respectively. CAT expression profile was 1.35-, 1.85-, 2.20-, and 3.45- folds in Foc, Foc + TN11, Foc + TN19, and Foc + TN11 + TN19 treatments, respectively as compared to control. Similarly, POD expression in Foc, Foc + TN11, Foc + TN19, and Foc + TN11 + TN19 treatments, respectively, were 1.29-, 1.52-, 1.51-, and 1.75-folds as compared to control.

### Effect of cell-free extracts and ethyl acetate extracts on fungal pathogens

Cell-free extract of *S. araujoniae* strains TN11 and TN19 were tested against all the four pathogens employing amendment with media at 1%. The highest growth inhibition of cell-free extract of TN11 was observed against *F. oxysporum* (45.55%), followed by *F. udum* (41.93%), *S. sclerotiorum* (38.33%), and *M. phaseolina* (33.33%). Similarly, cell-free extract of TN19 inhibited the growth of *F. udum*, *M. phaseolina*, *F. oxysporum*, and *S. sclerotiorum* by 45.45, 45, 44.9, and 38.3%, respectively.

**Figure 4:**
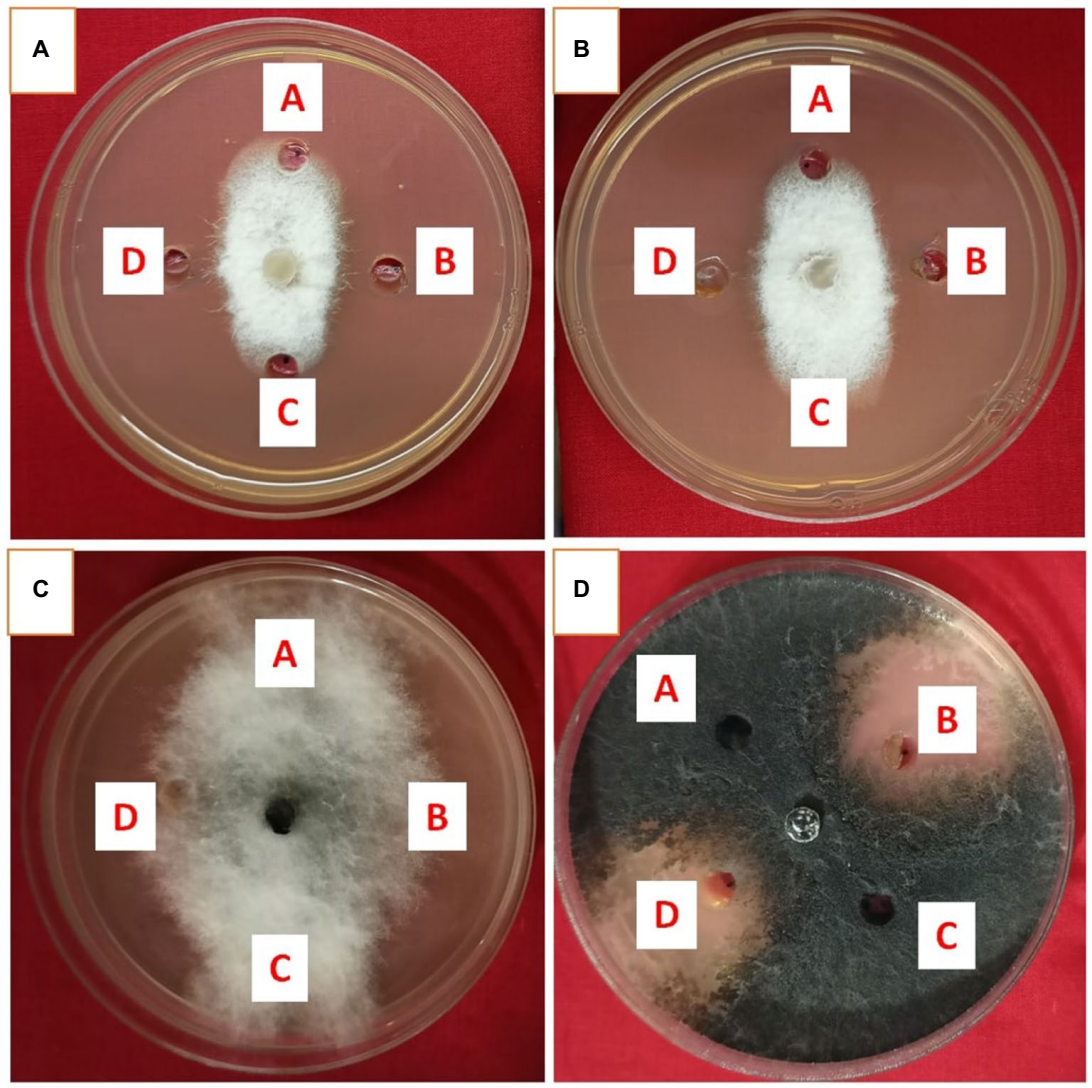
Antifungal activity of ethyl acetate extract of *Streptomyces araujoniae* TN11 in well diffusion assay against fungal pathogens *Fusarium udum* **(A)**, *Fusarium oxysporum* **(B)**, *Macrophomina phaseolina* **(C)** and *Sclerotinia sclerotiorum* **(D)**. Well A = Control, C = solvent control, B and D = ethyl acetate extract (20 μl).

**Figure 5:**
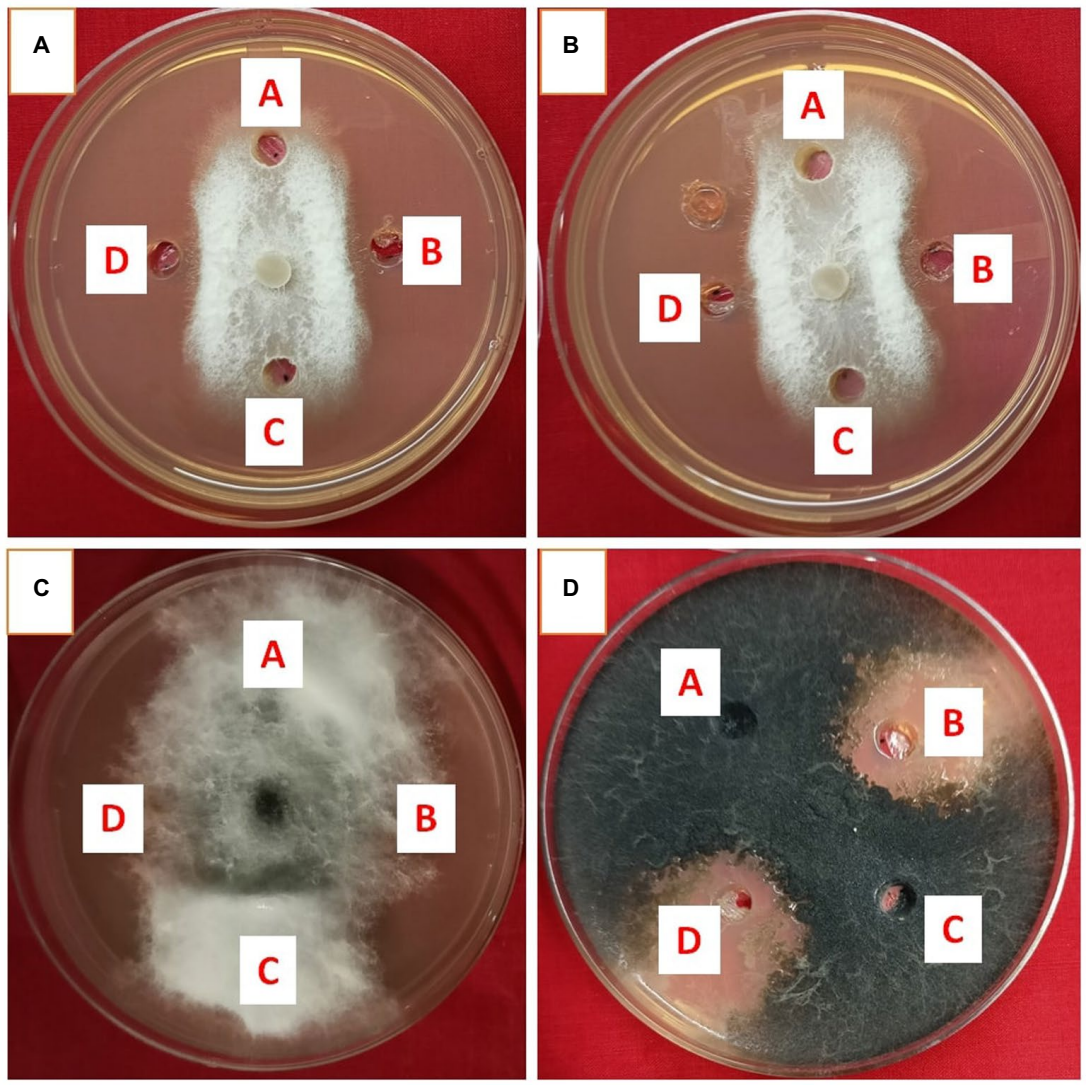
Antifungal activity of ethyl acetate extract of *Streptomyces araujoniae* TN19 in well diffusion assay against fungal pathogens *Fusarium oxysporum* **(A)**, *Fusarium udum* **(B)**, *Macrophomina phaseolina* **(C)** and *Sclerotinia sclerotiorum* **(D)**. Well A = Control, C = solvent control, B and D = ethyl acetate extract (20 μl).

**Figure 6:**
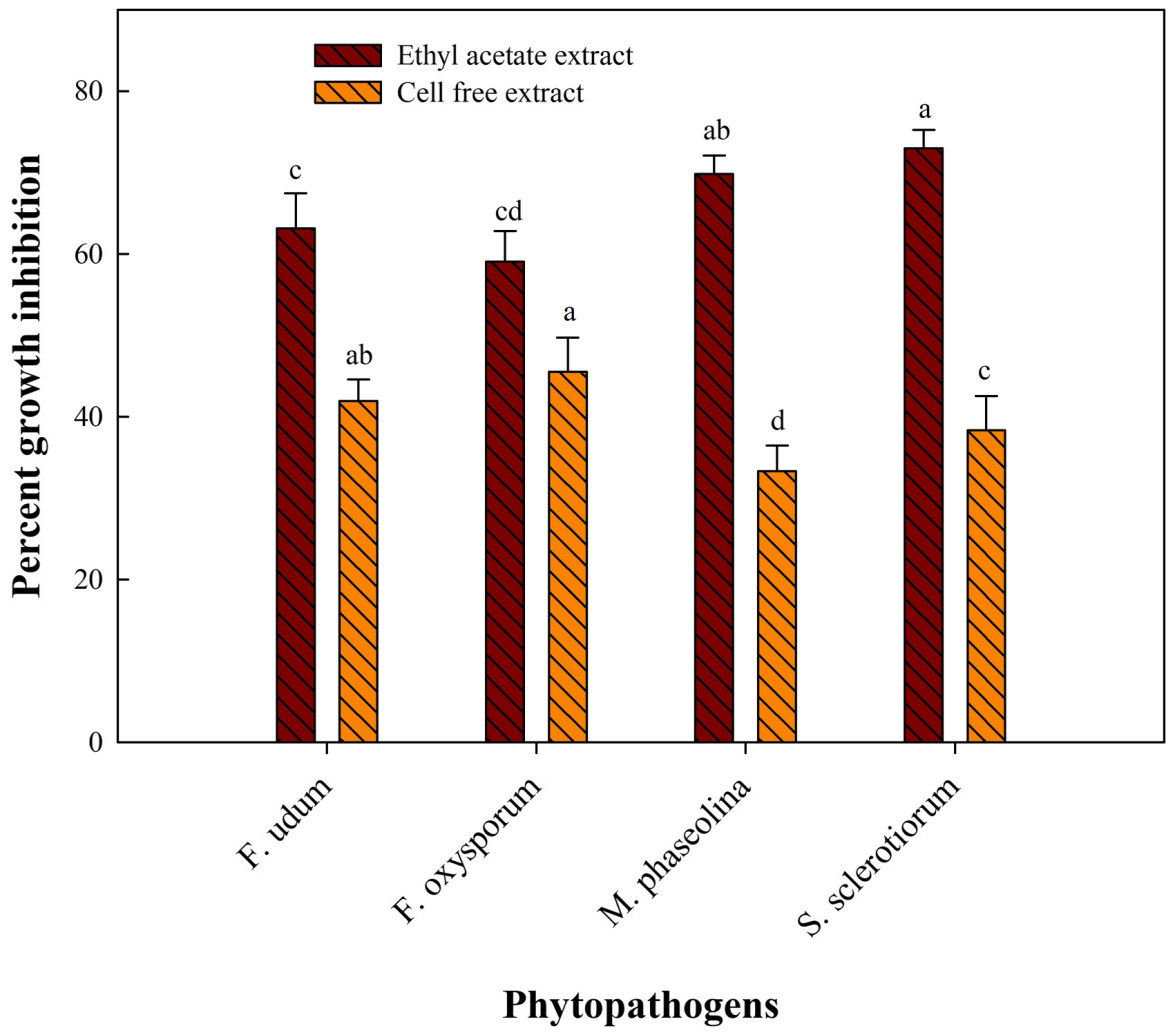
Antifungal activity (% inhibition) of *Streptomyces araujoniae* TN11 metabolites extracted in ethyl acetate and cell-free extract against different fungal pathogens. The data are the mean of three replicates ± standard error, within each graph, values followed by the same letter are not significantly different (*p* ≤ 0.05) according to Duncan’s multiple range test.

**Figure 7:**
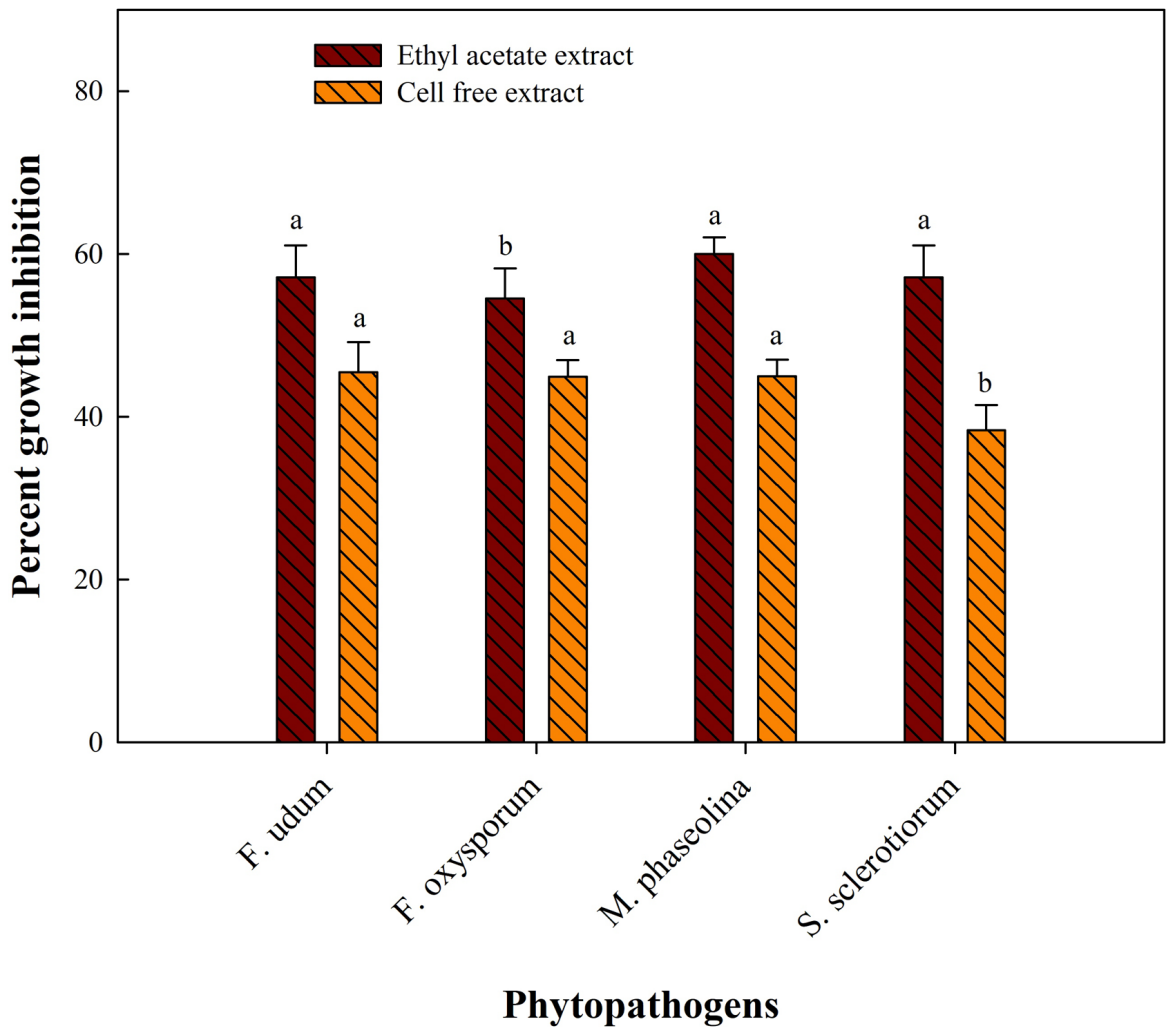
Antifungal activity (% inhibition) of *Streptomyces araujoniae* TN19 metabolites extracted in ethyl acetate and cell-free extract against different fungal pathogens. The data are the mean of three replicates ± standard error, within each graph, values followed by the same letter are not significantly different (*p* ≤ 0.05) according to Duncan’s multiple range test.

Metabolites extracted using different solvents ethyl acetate, n-hexane, and dimethyl sulfoxide were tested against all the four selected phytopathogens. Results revealed that extracts obtained using ethyl acetate were able to control the growth of all the pathogens tested while the extracts obtained using the other two solvents were not found to control the growth of any phytopathogen. Ethyl acetate extract of *S. araujoniae* TN11 showed the highest antagonistic activity in *S. sclerotiorum* (73.01%), followed by *M. phaseolina* (69.84%), *F. udum* (63.15%), and *F. oxysporum* (59.1%), while the extracts of *S. araujoniae* TN19 showed maximum antagonistic effect (60%) against *M. phaseolina* followed by *F. udum* (57.14%), *S. sclerotiorum* (57.14%), and *F. oxysporum* (54.54%; Figures 4–7).

### LC–MS of ethyl acetate extracts of *Streptomyces araujoniae* TN11 and TN19

Metabolites of *S. araujoniae* TN11 and TN19 extracted in ethyl acetate were subjected to LC–MS analyses. A total of more than 10 thousand compounds were detected through LC–MS in the ethyl acetate extracts of each strain. Ten major compounds detected in the extract with known antifungal properties are presented in Tables 3, 4. Erucamide was the most dominant antifungal compound found, followed by dinactin and valinomycin in the extracts of TN11. The extracts of TN19 showed 2-Octadecylfuran as the most dominant antifungal compound followed by valinomycin and dinactin. LC–MS studies revealed that *S. araujoniae* TN11 and TN19 produced a combination of antifungal compounds capable of inhibiting the development of several phytopathogenic fungi.

**Table 3:** List of some bioactive molecules with reported antifungal activity identified through LC–MS analyses of ethyl acetate extracts of *Streptomyces araujoniae* (TN11).

Sl. no.	Metabolites	Molecular formula	Retention time (min)	Peak area (%)	Activity against fungal pathogens	References
1	Erucamide	C_22_H_43_NO	26.321	7.970	*Fusarium oxysporum* f. sp. *cubense*	Qi et al., 2022
2	Dinactin	C_42_H_68_O_12_	24.447	3.920	*Colletotrichum gloeosporioides*, *Colletotrichum musae*, *Botryodiplodia theobromae*, *F. oxysporum* f. sp. *cubense*, *Magnaporthe oryzae*, *Alternaria solani*	Islam et al., 2016; Zhang et al., 2020
3	Valinomycin	C_54_H_90_N_6_O_18_	25.429	2.736	*Botrytis cinerea*, *Rhizoctonia solani*	Park et al., 2008; Jeon et al., 2019
4	4-Aminobenzoic acid	C_7_H_7_NO_2_	4.943	0.726	*M. oryzae, R. solani, Sclerotinia sclerotiorum*	Laborda et al., 2018
5	Docosanamide	C_22_H_45_NO	28.152	0.246	*C. gloeosporioides*, *B. cinerea, Colletotrichum acutatum*, *R. solani*	Vázquez-Fuentes et al., 2021
6	3-BHA	C_11_H_16_O_2_	1.194	0.294	*Aspergillus niger, Aspergillus oryzae*	Tabti et al., 2014
7	Tramadol	C_16_ H_25_ N O_2_	12.598	0.279	*Candida albicans*, *Tricophyton rubrum*	Kathwate and Karuppayil (2016)
8	(4aR,5R,6R,8aR)-2-(6,10-Dimethyl-2-undecanyl)-5,8a-dimethyldecahydro-6-isoquinolinol	C_24_H_47_NO	28.151	0.259	*Candida krusei*	Dos Reis et al., 2019
9	Maltol	C_6_H_6_O3	0.990	0.097	*Phytophthora capsici*, *Bursaphelenchus xylophilus*	Tian et al., 2020
10	Caryophyllene oxide	C_15_H_24_O	10.907	0.093	*Fusarium moniliforme, R. solani*, *Helminthosporium oryzae* and *A. solani*	Huang et al., 2019; Jassal et al., 2021

BHA-Butylated hydroxyanisole.

**Table 4:** List of some bioactive molecules with reported antifungal activity identified through LC–MS analyses of ethyl acetate extracts of *Streptomyces araujoniae* (TN19).

Sl. no.	Metabolites	Molecular formula	Retention time (min)	Peak area (%)	Activity against fungal pathogens	References
1	2-Octadecylfuran	C_22_H_40_O	26.302	7.998	*C. albicans*, *A. niger*	El-Sayed et al., 2020
2	Valinomycin	C_54_H_90_N_6_O_18_	25.402	3.887	*B. cinerea*, *R. solani*,	Park et al., 2008; Jeon et al., 2019
3	Dinactin	C_42_H_68_O_12_	24.370	1.560	*C. gloeosporioides*, *C. musae, B. theobromae*, *F. oxysporum* f. sp. *cubense*, *M. oryzae*, *A. solani*	Islam et al., 2016; Zhang et al., 2020
4	4-Aminobenzoic acid	C_7_H_7_NO_2_	4.851	1.310	*M. oryzae, R. solani, S. sclerotiorum*	Laborda et al., 2018
5	Diethylene glycol	C_4_H_10_O_3_	0.809	0.564	*C. albicans*, *C. glabrata*, C. *krusei*, *C. parapsilosis*, *A. flavus*, *A. nidulans*, *A. terreus*	Chandrika et al., 2016
6	3-BHA	C_11_H_16_O_2_	1.194	0.402	*A. niger, A. oryzae*	Tabti et al., 2014
7	Docosanamide	C_22_H_45_NO	28.170	0.275	*C. gloeosporioides*, *B. cinerea, C. acutatum*, *Fusarium* sp., *R. solani*	Vázquez-Fuentes et al., 2021
8	10-Undecenoic acid	C_11_H_20_O_2_	9.855	0.32530	*C. albicans*	Shi et al., 2016
9	Bengamide Z	C_18_H_32_N_2_O_7_	11.533	0.261	*C. albicans*	Jamison et al., 2019
10	TBHQ	C_10_H_14_O_2_	0.968	0.186	*Fusarium*, *Penicillium* and *Aspergillus*	Thompson (1992)

BHA-Butylated hydroxyanisole; TBHQ-tert-Butylhydroquinone.

## Discussion

Fungal phytopathogens pose a significant threat to agriculture, as they cause a variety of plant diseases (Sharma and Manhas, 2020) that cause severe damage, resulting in lower productivity. According to the food and agriculture organization, averting fungal diseases in five major crops, including maize, wheat, rice, potato, and soybean would be able to additionally feed 8.5% of the world’s population (Fisher et al., 2012). Fungal plant pathogens like *Sclerotinia*, *Fusarium*, *Macrophomina*, *Sarocladium, Rhizoctonia*, and *Aspergillus* have been found to cause several dreaded diseases in many food, feed, and cash crops (Derbyshire and Denton-Giles, 2016; Hassan et al., 2021).

The *Fusarium oxysporum* f. sp. *ciceris* (Foc) that causes chickpea wilt was first discovered in India in 1918 and is currently widespread in many nations. The pathogen exhibits a wide range of cultural traits and pathogenicity with as many as eight pathogenic races identified (Ankati et al., 2021). Yield losses range from 10 to 100% depending on the vulnerability of the variety and the agroclimatic conditions (Chand and Khirbat, 2009). Overuse of chemical fungicides for control of plant diseases is a very common practice in most developing nations which drastically affects the ecosystem and health. Generally, chemical pesticides persist in the environment and devastate the useful soil microbes that have plant growth-promoting traits and maintain the fertility of the soil. Microbes and their secondary metabolites are more eco-friendly and are sustainable alternatives to insecticides and herbicides. Actinomycetes are widely known among microbes for the production of secondary metabolites with antimicrobial properties (Shahid et al., 2021). More than 70% of the well-recognized secondary metabolites are products of actinomycetes; among them over 10,000 bioactive compounds have been reported from *Streptomyces* alone. *Streptomyces* sp. displayed a broad range of antagonistic activity against numerous fungal pathogens, which include *Fusarium oxysporum, Rhizoctonia solani*, *Colletotrichum gloeosporioides*, *Botrytis cinerea*, *Cochliobolus miyabeanus*, and *Pyricularia oryzae*. Recently Ankati et al. (2021) reported *Streptomyces griseus* strains (CAI-24, CAI-121, and CAI-127), *Streptomyces coelicolor* KAI-90, and *Streptomyces africanus* KAI-32 with properties of biocontrol against Foc mediated wilt and plant growth promotion in chickpea. Screening of effective antagonistic actinomycetes is essential for the development of various biocontrol agents; *Streptomyces* sp. SCA3-4 demonstrated wide antifungal potential against 13 fungal pathogens (Qi et al., 2019). Sarika et al. (2021) reported the antibacterial and antifungal activity of *Streptomyces felleus* BHPL-KSKU5 isolated from a coal mine in Telangana (India). In the present study ethyl acetate extracts of the strains, *Streptomyces araujoniae* TN11 and TN19 showed antagonistic activity against *M. phaseolina*, *F. oxysporum*, *F. udum,* and *S. sclerotiorum* fungal pathogens. Similarly, *Streptomyces* sp. M4 isolated from soil (Punjab, India) and secondary metabolites extracted in ethyl acetate showed antifungal activity against various pathogens like *Botrytis cinerea*, *Fusarium* spp., *Cladosporium herbarum, Colletotrichum* spp., and *Alternaria* spp. (Sharma and Manhas, 2020).

Plants are constantly subjected to diverse abiotic and biotic stressors in their natural environment. However, they show a high level of phenotypic flexibility and adaptability, which is defined by the plant’s genome, to respond to such stimuli (Aamir et al., 2017). By engaging complex, synergistic, and/or antagonistic signaling networks plants respond effectively to such stressors (Pandey and Somssich, 2009). Microorganisms that support plant growth and biocontrol pests have become secure substitutes for chemical pesticides. Several bacterial and fungal antagonists have been discovered for the biological control of *Fusarium* wilt in chickpea like *Trichoderma* spp., *Pseudomonas* spp., *Bacillus* spp., isolates of non-pathogenic *Fusarium oxysporum,* and *Streptomyces* spp. (Jiménez-Díaz et al., 2015; Ankati et al., 2021). The genus *Streptomyces* shows great promise for fostering plant growth and defending plants from several diseases. A variety of bacterial and fungal phytopathogens can be effectively controlled by *Streptomyces araujoniae* and their metabolites (Silva et al., 2014). In our present study bio-priming with *Streptomyces araujoniae* strains, TN11 and TN19 reduce the disease development by Foc in chickpea (variety Pusa 362) as suggested by the disease severity index.

Reduction in disease development may be attributed to antifungal compounds produced by TN11 and TN19. Antagonism of *Streptomyces* spp. against fungi is generally mediated *via* the synthesis of antimicrobial secondary metabolites, the release of lytic and cell wall disintegrating enzymes, and competition for resources (Baz et al., 2012). The synthesis of antimicrobial compounds by the genus *Streptomyces* has previously been documented and has been the subject of extensive research in recent years (Santoyo et al., 2021). *Streptomyces* species generate a variety of bioactive compounds with antibacterial properties. For instance, *S. padanus* PMS-702 synthesizes the polyene macrolide antibiotic fungichromin, which exhibits strong antagonistic properties against many phytopathogenic fungi (Fan et al., 2019). Hyphae development in *Alternaria brassicicola* was restricted by 6-prenylindole; a compound produced by *Streptomyces* sp. TP-A0595 (Singh and Dubey, 2018). In the present study, through LC–MS we identified 10 major compounds produced by TN11 and TN19 with reported antifungal properties. Erucamide, dinactin, valinomycin, and strophanthidol were major compounds identified in the TN11 strain with peak areas, of 7.97, 3.92, 2.74, and 2.39%, respectively. Similarly, the TN19 strain had predominantly valinomycin (3.89%), dinactin (1.56%), and 4-aminobenzoic acid (1.31%). Earlier Qi et al. (2022) and Xie et al. (2021), reported antifungal properties of Erucamide. Antifungal properties of dinactin have been reported against various fungi like *Pestolotiopsis mangiferae*, *Neoscytalidium dimidiatum*, *Fusarium dimerum*, *Magnaporthe oryzae*, *Alternaria solani*, *Fusarium* oxysporum f.sp. *cubense*, *Colletotrichum gloeosporioides*, and *Colletotrichum musae* (Islam et al., 2016; Zhang et al., 2020). Similarly, Jeon et al. (2019), observed antifungal activities of valinomycin against *Botrytis cinerea*, *Rhizoctonia solani*, and *C. gloeosporioides*. The findings of our study demonstrated the antifungal properties of TN11 and TN19 strains and suggest their implementation as biocontrol agents.

The effect of bio-priming in controlling the disease is more pronounced in consortium treatment as compared to individual treatments. The synergistic effect of the consortium of TN11 and TN19 might be due to the complementation of the antifungal compounds produced by them individually. Earlier synergistic effects of different consortia on various crops have been reviewed by Santoyo et al. (Santoyo et al., 2021). Bio-priming with TN11 and TN19 also improved plant growth in presence of the Foc challenge. Like the reduction in disease development, a synergistic effect by the consortium is shown in plant growth parameters too. As compared to individual strains’ treatment consortium treatment resulted in a more pronounced enhancement of plant growth. Such enhanced performance by consortium over the individual strain treatments has been reported by Singh et al. (Singh et al., 2014). For commercial applications, the microbial consortium has been suggested as a sustainable method for improving plant health and growth (Kofoworola et al., 2016). Better plant growth parameters observed in the presence of Foc challenge for treatments receiving priming with TN11, and TN19 individually and as a consortium suggest that these strains reduce the negative effect of the pathogen on plants. *Streptomyces* spp. being a member of the rhizospheric microbial community got the ability to act as a plant growth promoter (Dias et al., 2017). Plant pathogens causing wilt hinders the transport of water within the plants thereby lowering RWC and inducing membrane damage; membrane damage in turn leads to increased EL (Rodrigues et al., 2018). In the present study, the application of the TN11 and TN19 strains results in significantly increased RWC and reduced EL by reducing disease severity; similar results were reported in maize and groundnut (Ghorai et al., 2015; Fazal and Bano, 2016) using different species of *Pseudomonas.*

When exposed to oxidative stress, plants produce H_2_O_2_ which plays a double function, as it may result in programmed cell death, but it can also serve as a quick signal to activate antioxidant defense responses (Gullner et al., 2018). In Foc + TN11, Foc + TN19, and Foc + TN11 + TN19 treated plants, H_2_O_2_ accumulations were lower compared to pathogen treated plants, signifying a feeble balance between H_2_O_2_ scavenging and H_2_O_2_ generation. The increased MDA concentration in the leaf is considered an oxidative stress indicator responsible for cellular damage (Lata et al., 2011). The reduction in MDA contents due to the Foc + TN11, Foc + TN19, and Foc + TN11 + TN19 treatment suggests it can reduce the peroxidation of plasma lemma under pathogen infection to shield the leaf cell membrane from damage. Similar results were reported in tomato plants inoculated with *Bacillus cereus* AR156 (Wang et al., 2013), in maize inoculated with *Pseudomonas putida* (Nosheen and Bano, 2014), and in rice inoculated with *P. fluorescens, P. jessenii,* and *P. synxantha* (Gusain et al., 2015). Proline protects plant cells from damage by acting as both an osmotic agent and a scavenger of reactive oxygen species (Liu et al., 2011). In the present study, proline level rises in Foc + TN11, Foc + TN19, and Foc + TN11 + TN19 treated plants. The elevated levels of proline within infected plant tissues can be interpreted as a host reaction to protect against stresses caused by pathogens (Rodrigues et al., 2018). Chlorophyll and carotenoids are two important leaf pigments that act as a physiological status to evaluate the performance of photosynthetic machinery under stress conditions either biotic or abiotic (Ansari et al., 2019). Biotic stress frequently causes leaves to exhibit chlorosis and necrosis because it reduces the number of chloroplasts and breaks down chlorophyll (Dinis et al., 2011), which was observed in the current study too. Chlorophyll and carotenoid content declined significantly in Foc challenged plants; the reduction was lower in Foc challenged plants primed with TN11, TN19, and TN11 + TN19.

All plants treated with Foc and the combination of Foc and TN11, TN19 showed differential SOD activity compared to the control unprimed plants. Foc challenged plants and Foc + TN11, Foc + TN19, Foc + TN11 + TN19, treated plants showed higher SOD activity compared to control plants. Plants have evolved a variety of defense mechanisms to cope with pathogen challenges, among them mechanism of ROS accumulation is the most significant which includes SOD accumulation (Aamir et al., 2019). Peroxidase is recognized as a major pathogen-related protein (PR-protein) or defense protein that participates in multiple physiological adaptations of plants against biotic stresses. Salla et al. (2016) reported that plants treated with *Streptomyces* as a biocontrol agent, exhibiting *Fusarium* wilt showed increased peroxidase activity in cucumber (Zhao et al., 2012). The antioxidant defense system of plants includes peroxidase and CAT, which scavenges ROS to quench the negative effects of stressors (Das and Roychoudhury, 2014). In this study, the combination of Foc + TN11, Foc + TN19, and Foc + TN11 + TN19, induces catalase activity compared to the control. Nikoo et al. (2014) demonstrated that *Pseudomonas fluorescens* treatment induces systemic resistance in tomatoes through defense-related enzymes POD and CAT. They demonstrated increased activity of POD and CAT due to inoculation of *P. fluorescens* and treatment with elicitors as compared to the activities of POD and CAT in plants treated with pathogen alone. Similar findings were also reported by Ankati et al. (2021), in chickpea plants after *Streptomyces* consortium treatment.

Enhanced expression of genes like *SOD*, *CAT,* and *POD* results in diminished ROS activity, which stimulates the activation of the different signaling cascades. In our study, like increased enzyme activity of SOD, CAT, and POD, enhanced expression of their respective genes was observed, and it was maximum in *Streptomyces araujoniae* consortium treated plants. Similarly, Mastouri et al. (2012) reported an enhanced expression of *SOD*, *CAT*, and *POD* genes because of *T. harzianum* treatment in tomatoes. Aamir et al. (2019), reported enhanced expression of these genes in tomato plants after bio-priming with *T. erinaceum*.

## Conclusion

The present study has shown that the application of *Streptomyces araujoniae* strains individually and as a consortium reduces wilt severity in chickpea plants. Reduction in disease development is supported by the production of antifungal metabolites at a higher level by TN11 and TN19. The results on disease development, plant growth parameters, physiological and biochemical parameters, and gene expression studies suggest that the consortium of TN11 and TN19 can act as an efficient biocontrol tool against chickpea wilt caused by Foc.

## Data availability statement

The original contributions presented in the study are included in the article/Supplementary material, further inquiries can be directed to the corresponding author.

## Author contributions

MK and ASa conceived the idea and planned the experiments. MZ, PT, WA, and SK performed the experiments. WA, MZ, and PT analyzed the data. WA, MK, and MZ wrote the manuscript. MK, HC, ASr, ASa, and US checked the final draft of the manuscript. All authors contributed to the article and approved the submitted version.

## Funding

Indian Soil Microbiome Project, funded by ICAR-NBAIM, Mau, India.

## Conflict of interest

The authors declare that the research was conducted in the absence of any commercial or financial relationships that could be construed as a potential conflict of interest.

## Publisher’s note

All claims expressed in this article are solely those of the authors and do not necessarily represent those of their affiliated organizations, or those of the publisher, the editors and the reviewers. Any product that may be evaluated in this article, or claim that may be made by its manufacturer, is not guaranteed or endorsed by the publisher.

## Footnotes

^2^ https://www.mzcloud.org/

^3^ http://www.chemspider.com
