# Supplementary material for: Bio-priming with a consortium of Streptomyces araujoniae strains modulates defense response in chickpea against Fusarium wilt
Source: Front Microbiol. 2022 Sep 8;13:998546. doi: 10.3389/fmicb.2022.998546 (PMC9493686; doi:10.3389/fmicb.2022.998546)
Supplement: Supplementary file 1 [file Table_1.DOCX]

Supplementary Table 1: Nucleotide sequences of primers used for reverse transcription quantitative PCR analysis.

| Gene names | Forward primers 5’ – 3’ | Reverse primers 5’ – 3’ |
| --- | --- | --- |
| *SOD* | CCATCCAGTTCGTCCAAGAT | GTATCGCCAAGAGCATGGAT |
| *POD* | CCTCCAAAGAATCCGTCGTA | TTGGCTTTGAGTGCATTGAG |
| *CAT* | TTCCATCACTGCAGCTTTGCTTCG | TTTGTTTGGGCCAGCTTCTCTCTC |
